# A prospective, multicenter, observational study of ixazomib plus lenalidomide-dexamethasone in patients with relapsed/refractory multiple myeloma in Japan

**DOI:** 10.1007/s00277-023-05428-7

**Published:** 2023-09-11

**Authors:** Yuichi Horigome, Masaki Iino, Yoriko Harazaki, Takahiro Kobayashi, Hiroshi Handa, Yasushi Hiramatsu, Taiga Kuroi, Kazuki Tanimoto, Kosei Matsue, Masahiro Abe, Tadao Ishida, Shigeki Ito, Hiromi Iwasaki, Junya Kuroda, Hirohiko Shibayama, Kazutaka Sunami, Hiroyuki Takamatsu, Hideto Tamura, Toshiaki Hayashi, Kiwamu Akagi, Takahiro Maeda, Takahiro Yoshida, Ikuo Mori, Tomohiro Shinozaki, Shinsuke Iida

**Affiliations:** 1 Department of Hematology, Kitasato University School of Medicine, Sagamihara, Japan; 2 Department of Hematology, Yamanashi Prefectural Central Hospital, Kofu, Japan; 3 Department of Hematology, Miyagi Cancer Center, Natori, Japan; 4 Department of Hematology, Nephrology and Rheumatology, Akita University Graduate School of Medicine, Akita, Japan; 5 Department of Hematology, Gunma University Graduate School of Medicine, Maebashi, Japan; 6 Department of Hematology and Oncology, Japanese Red Cross Society Himeji Hospital, Himeji, Japan; 7 Department of Hematology, Chugoku Central Hospital, Fukuyama, Japan; 8 Department of Hematology and Oncology, Japanese Red Cross Fukuoka Hospital, Fukuoka, Japan; 9 Division of Hematology/Oncology, Department of Internal Medicine, Kameda Medical Center, Kamogawa, Japan; 10 Department of Hematology, Endocrinology and Metabolism, Tokushima University Graduate School, Tokushima, Japan; 11 Department of Hematology, Japanese Red Cross Medical Center, Tokyo, Japan; 12 Department of Hematology and Oncology, Iwate Medical University Hospital, Iwate, Japan; 13 Department of Hematology, National Hospital Organization Kyushu Medical Center, Fukuoka, Japan; 14 Division of Hematology and Oncology, Kyoto Prefectural University of Medicine, Kyoto, Japan; 15 Department of Hematology, National Hospital Organization Osaka National Hospital, Osaka, Japan; 16 Department of Hematology, National Hospital Organization Okayama Medical Center, Okayama, Japan; 17 Department of Hematology, Kanazawa University Hospital, Kanazawa, Japan; 18 Department of Hematology, Nippon Medical School, Tokyo, Japan; 19 Department of Hematology, Teine Keijinkai Hospital, Sapporo, Japan; 20 Division of Molecular Diagnosis and Cancer Prevention, Saitama Cancer Center, Ina, Japan; 21 Division of Precision Medicine, Kyushu University Graduate School of Medical Sciences, Fukuoka, Japan; 22 Medical Affairs, Japan Oncology Business Unit, Takeda Pharmaceutical Co. Ltd, Tokyo, Japan; 23 Department of Information and Computer Technology, Faculty of Engineering, Tokyo University of Science, Tokyo, Japan; 24 Department of Hematology and Oncology, Nagoya City University Institute of Medical and Pharmaceutical Sciences, 1, Kawasumi, Mizuho-Cho, Mizuho-Ku, Nagoya, Aichi 467-8601 Japan

**Keywords:** Effectiveness, Ixazomib, Multiple myeloma, Relapsed/refractory, Real-world data, Safety

## Abstract

Real-world studies permit inclusion of a more diverse patient population and provide more information on the effectiveness of treatments used in routine clinical practice. This prospective, multicenter, observational study investigated the effectiveness and safety of ixazomib plus lenalidomide and dexamethasone (IRd) in 295 patients with relapsed/refractory multiple myeloma (RRMM) in routine clinical practice in Japan. Patients had a median age of 74 years, 80.0% were aged ≥ 65 years, 42.0% had received ≥ 3 lines of prior treatment, and 28.5% were “frail” according to the International Myeloma Working Group frailty score. After a median follow-up of 25.0 months, median progression-free survival (PFS) was 15.3 (95% CI 12.4–19.5) months, while median overall survival was not reached. The overall response rate was 53.9%, and 31.5% of patients had a very good partial response or better. In the subgroup analysis, median PFS was better in patients with 1 versus 2 or ≥ 3 lines of prior treatment (29.0 vs 19.2 or 6.9 months) and paraprotein versus clinical relapse (16.0 vs 7.9 months), but median PFS was not notably affected by frailty score or age group. Dose adjustment was more frequent among patients aged > 75 years, especially early after IRd treatment initiation. Treatment-emergent adverse events (TEAEs) of any grade occurred in 84.4% of patients and 24.7% of patients discontinued treatment due to TEAEs; no new safety concerns were found. These findings suggest that oral IRd triplet regimen is an effective and tolerable treatment option for RRMM patients in real-world settings outside of clinical trials.

ClinicalTrials.gov identifier: NCT03433001; Date of registration: 14 February 2018.

**Supplementary Information:**

The online version contains supplementary material available at 10.1007/s00277-023-05428-7.

## Introduction

According to the Global Cancer Observatory report, the number of new cases of multiple myeloma (MM) in Japan in 2020 was 7,234 and the 5-year prevalence rate was 15.2 per 100,000 persons [1]. The median age at diagnosis is 69 years, and the incidence of MM is expected to increase as society ages [2]. Moreover, with a rapidly aging population, the number of elderly and frail MM patients is increasing in Japan [3].

In recent years, the availability of drugs with novel mechanisms of action, such as proteasome inhibitors (PIs), immunomodulators and anti-CD38 monoclonal antibodies, has considerably improved therapeutic outcomes [4–6]. Ixazomib is an oral PI [7, 8], which in combination with lenalidomide and dexamethasone (hereafter referred to as IRd) was approved in Japan for the treatment of relapsed/refractory MM (RRMM) in 2017 [9]. This approval was based on the results of the phase III TOURMALINE-MM1 trial, which included 41 Japanese patients [10]. Compared with placebo in combination with lenalidomide and dexamethasone (Rd), IRd demonstrated superior progression-free survival (PFS; median 20.6 vs 14.7 months; hazard ratio [HR] = 0.74; *p* = 0.01) and a higher overall response rate (ORR; 78% vs 72%; *p* = 0.04) as well as a higher rate of patients experiencing, as a minimum, a very good partial response rate (≥ VGPR; 48% vs 39%; *p* = 0.01), with limited additional toxicity [10].

However, treatment outcomes in routine clinical practice are often poorer than those in randomized clinical trials of MM therapy [11]. In part, this could result from the fact that up to 72% of real-world patients with RRMM do not meet the eligibility criteria for clinical trials [12]. Real-world studies with less stringent eligibility criteria may include a more diverse population of RRMM patients and provide more information on the effectiveness of treatments used in routine clinical practice.

Real-world data on the use of IRd in Japanese RRMM patients are limited. Therefore, a prospective, observational study was conducted to investigate the effectiveness and safety of IRd therapy in patients with RRMM in routine clinical practice in Japan.

## Methods

### Study design

This was a non-interventional, multicenter, prospective, observational study conducted in Japan in patients with RRMM who were treated with IRd. The study was registered at ClinicalTrials.gov (identifier: NCT03433001) on 14 February 2018 and the Japan Pharmaceutical Information Center – Clinical Trials Information (identifier: JapicCTI-183860) on 09 February 2018. Patients were enrolled at 81 sites in Japan between April 2018 and May 2019. The observation period for each patient was from the start of IRd therapy until either 24 months after the enrollment date of the final patient, or until death or withdrawal of consent, whichever was earlier.

This study was conducted in accordance with the ethical principles of the Declaration of Helsinki and the Ethical Guidelines for Medical and Health Research Involving Human Subjects, and complied with all applicable laws and regulations, including data privacy laws, and guidelines and regulations on conflicts of interest. Written informed consent was obtained from all patients prior to any study procedures being undertaken.

### Patient selection and treatment

The study enrolled adult (age ≥ 20 years) patients with RRMM scheduled to start IRd therapy, including those who were refractory to prior lenalidomide or PI-based therapy, while patients with a history of previous treatment with ixazomib were excluded (see Supplementary Table S1 for complete inclusion/exclusion criteria). Patient care and evaluations were determined by the treating physicians.

Patients received IRd treatment in accordance with the Japanese package insert of each individual drug [13–15]. As such, the recommended starting dose of each drug, administered as a 28-day treatment cycle was: ixazomib 4 mg once weekly administered orally on Days 1, 8, and 15 [14]; lenalidomide 25 mg/day, administered on Days 1 through 21 [13]; and dexamethasone 40 mg once weekly, administered on Days 1, 8, 15, and 22 [15]. However, the starting dose for lenalidomide was adjusted according to patients’ baseline renal function (i.e., creatinine clearance value) [13]. Treatment was continued until disease progression or the development of unacceptable toxicity, or patient/physician decision to end treatment.

### Endpoints

The primary endpoint was PFS. Secondary endpoints were: PFS rates at 12 and 24 months; overall survival (OS); best response; ORR; time to next treatment (TTNT); duration of therapy (DOT); duration of response (DOR); proportion of patients continuing treatment at 12 and 24 months; rate of minimal residual disease (MRD) negativity at complete response (CR); relative dose intensity (RDI) for ixazomib, lenalidomide, and dexamethasone; health-related quality of life (HRQoL), evaluated by patient-reported instruments including the European Organisation for Research and Treatment of Cancer Quality of Life Questionnaire-Core 30 module (EORTC QLQ-C30) and the myeloma-specific module (EORTC QLQ-MY20); and assessment of the severity, frequency, and incidence of any treatment-emergent adverse events (TEAEs; for additional information on definitions of endpoints and assessments, see the Supplementary Methods). Subgroup analyses were also conducted by age, frailty score, number of prior regimens, type of relapse (clinical or paraprotein) before IRd, and cytogenetic risk [t(4;14), t(14;16), t(11;14), del(17p), or 1q21 gain]. Clinical relapse was defined as disease recurrence with CRAB (i.e., **c**alcium elevation, **r**enal insufficiency, **a**nemia, and **b**one abnormalities) symptoms, and paraprotein relapse as disease recurrence with elevated M-protein levels but without CRAB symptoms. Cytogenetic risks were defined as follows: high-risk was the presence of ≥ 1 of t(4;14), t(14;16), or del(17p); standard-risk was the absence of high-risk cytogenetic abnormalities; expanded high-risk was the presence of ≥ 1 of t(4;14), t(14;16), or del(17p), and/or 1q21 gain; and modified standard-risk was the absence of expanded high-risk cytogenetic abnormalities.

### Statistical analysis

The planned sample size was 300 patients. Assuming that IRd therapy would have a similar median PFS (the primary outcome) as reported for Rd therapy in Japanese patients with RRMM (15–18 months) [10, 16–18] and that there would be no difference in the relative effectiveness of IRd versus Rd in this study versus the TOURMALINE-MM1 study [10], we estimated that a population of 300 patients would be required to achieve a PFS of 18.8–22.5 months with an accuracy of ± 3 months. Data were analyzed in the full analysis set (FAS), which comprised all enrolled patients who received at least one dose of ixazomib, and the safety analysis set, which comprised all patients who received at least one dose of any drug used in IRd therapy (i.e., ixazomib, lenalidomide, or dexamethasone). Baseline patient characteristics and response and safety data were summarized using descriptive statistics. PFS, OS, TTNT, and DOR were estimated for the FAS using the Kaplan–Meier method. The two-sided 95% confidence intervals (CIs) for ORR and MRD negativity were calculated based on a binomial distribution. Summary statistics for the RDI of ixazomib, lenalidomide, and dexamethasone were calculated in the safety analysis set. For evaluating the association of PFS with patient baseline factors of interest, univariate and multivariate Cox proportional hazards regression analyses were used to determine HRs and associated 95% CIs. All statistical analyses were conducted with SAS version 9.4.

## Results

### Patient background

A total of 295 patients with RRMM who had received at least one cycle of therapy with IRd were included in this analysis and comprised the FAS. Patient demographics, disease characteristics, and prior treatment exposure are described in Table 1. Patients had a median age of 74 years and 38.6% were aged > 75 years. Mean time from initial diagnosis was 46.1 months. The most common type of myeloma was immunoglobulin G (IgG) type (50.8% of patients). At study entry, 70.5% of patients were International Staging System stage I or II (10.5% were stage III and data for 19.0% of patients were missing), and 15.6% had an Eastern Cooperative Oncology Group (ECOG) performance status of ≥ 2. Additionally, 37.3% of patients had a creatinine clearance of < 60 mL/min, including 11.6% with a creatinine clearance of < 30 mL/min, and more than half of the study population were “frail” or of “intermediate fitness” according to the International Myeloma Working Group (IMWG) frailty score. Moreover, 23.1% of patients had high-risk cytogenetic abnormalities and 50.2% had expanded high-risk cytogenetic abnormalities. Cytogenetic abnormalities included t(4;14) in 11.9% of patients, t(14;16) in 1.7%, t(11;14) in 12.9%, del(17p) in 15.3%, and 1q21 gain in 42.0% of patients. The number of patients with double or triple cytogenetic abnormalities is given in Supplementary Table S2.

**Table 1:** Baseline demographics and disease characteristics in patients with RRMM included in the full analysis set

	***N* =** 295
Age, median (range), years	74 (43–90)
≤ 65, *n* (%)	59 (20.0)
> 65 to ≤ 75, *n* (%)	122 (41.4)
> 75, *n* (%)	114 (38.6)
Male sex, *n* (%)	168 (56.9)
Type of myeloma, *n* (%)	
IgG type	150 (50.8)
IgA type	69 (23.4)
Bence Jones type	62 (21.0)
Other	14 (4.7)
Time since initial diagnosis, median (range), months	35.0 (0.6–240.2)
ECOG performance status, *n* (%)	
0 or 1	237 (80.3)
2	23 (7.8)
≥ 3	23 (7.8)
Missing	12 (4.1)
ISS stage (at IRd initiation), *n* (%)	
Stage I or II	208 (70.5)
Stage III	31 (10.5)
Missing	56 (19.0)
Creatinine clearance, mL/min, *n* (%)	
< 60	110 (37.3)
≥ 60	167 (56.6)
Missing	18 (6.1)
IMWG frailty score, *n* (%)^a^	
Frail	84 (28.5)
Intermediate fitness	72 (24.4)
Fit	124 (42.0)
Missing	15 (5.1)
Disease status, *n* (%)	
Clinical relapse^b^	69 (23.4)
Paraprotein relapse^c^	156 (52.9)
Other	70 (23.7)
Cytogenetic risk, *n* (%)^d^	
High-risk^e^	68 (23.1)
Standard risk^f^	156 (52.9)
Expanded high-risk^g^	148 (50.2)
Modified standard risk^h^	76 (25.8)
Missing	71 (24.1)
Prior treatment regimens, median (range), months	2.0 (1.0–12.0)
1, *n* (%)	90 (30.5)
2, *n* (%)	81 (27.5)
3, *n* (%)	63 (21.4)
≥ 4, *n* (%)	61 (20.7)
Prior stem cell transplant, *n* (%)	91 (30.8)
Prior proteasome inhibitor therapy, *n* (%)	
Bortezomib	226 (76.6)
Carfilzomib	38 (12.9)
Prior immunomodulatory drug therapy, *n* (%)	
Lenalidomide	244 (82.7)
Pomalidomide	38 (12.9)
Prior daratumumab therapy, *n* (%)	26 (8.8)

*ECOG* Eastern Cooperative Oncology Group, *Ig* immunoglobulin, *IMWG* International Myeloma Working Group, *IRd* ixazomib + lenalidomide + dexamethasone, *ISS* International Staging System, *RRMM* relapsed/refractory multiple myeloma, *SD* standard deviation

^a^Patients were assessed on the activities of daily living and instrumental activities of daily living items on the IMWG frailty scale; Charlson Comorbidity Index items were also assessed

^b^Clinical relapse was defined as disease recurrence with CRAB (i.e., **c**alcium elevation, **r**enal insufficiency, **a**nemia, and **b**one abnormalities) symptoms

^c^Paraprotein relapse was defined as disease recurrence with elevated M-protein levels but without CRAB symptoms

^d^Cut-off levels were 5% positive cells for del(17p)) and 3% for t(4;14), t(11;14), t(14;16), and 1q21 gain

^e^High-risk was defined as the presence of ≥ 1 of t(4;14), t(14;16), or del(17p)

^f^Standard risk was defined as the absence of high-risk cytogenetic abnormalities

^g^Expanded high-risk was defined as the presence of ≥ 1 of t(4;14), t(14;16), or del(17p) and/or 1q21 gain

^h^Modified standard risk was defined as the absence of expanded high-risk cytogenetic abnormalities

Before study entry, 42.0% of patients had received ≥ 3 lines of prior treatment (Table 1). The proportion of patients with 1, 2 and ≥ 3 prior regimens among patients with high-risk cytogenetic abnormalities were 23.5%, 27.9%, and 48.5%, respectively. In contrast, the proportion of patients with 1, 2, and ≥ 3 prior regimens among patients with standard-risk cytogenetic abnormalities were 32.7%, 28.8%, and 38.5%, respectively. The most common regimen received at any line of treatment prior to initiating IRd was lenalidomide + dexamethasone (52.5% of patients), followed by bortezomib + dexamethasone (36.9%) and bortezomib + lenalidomide + dexamethasone (20.0%; Supplementary Table S3). The most common regimens received in the treatment line immediately prior to IRd initiation were lenalidomide + dexamethasone (30.5% of patients), bortezomib + lenalidomide + dexamethasone (9.8%), and bortezomib + dexamethasone (9.5%; Supplementary Table S4).

### Treatment regimen

In cycle 1 of treatment, 290 patients (98.3%) received IRd, while others received ixazomib + lenalidomide (*n* = 3; 1.0%), ixazomib + dexamethasone (*n* = 1; 0.3%), or ixazomib monotherapy (*n* = 1; 0.3%). Of the five patients who did not receive IRd in cycle 1, two patients (who had received ixazomib + lenalidomide) dropped out in cycle 1, while the other three received IRd therapy at the appropriate time in cycle 2 or later.

### Treatment exposure

**Fig. 1:**
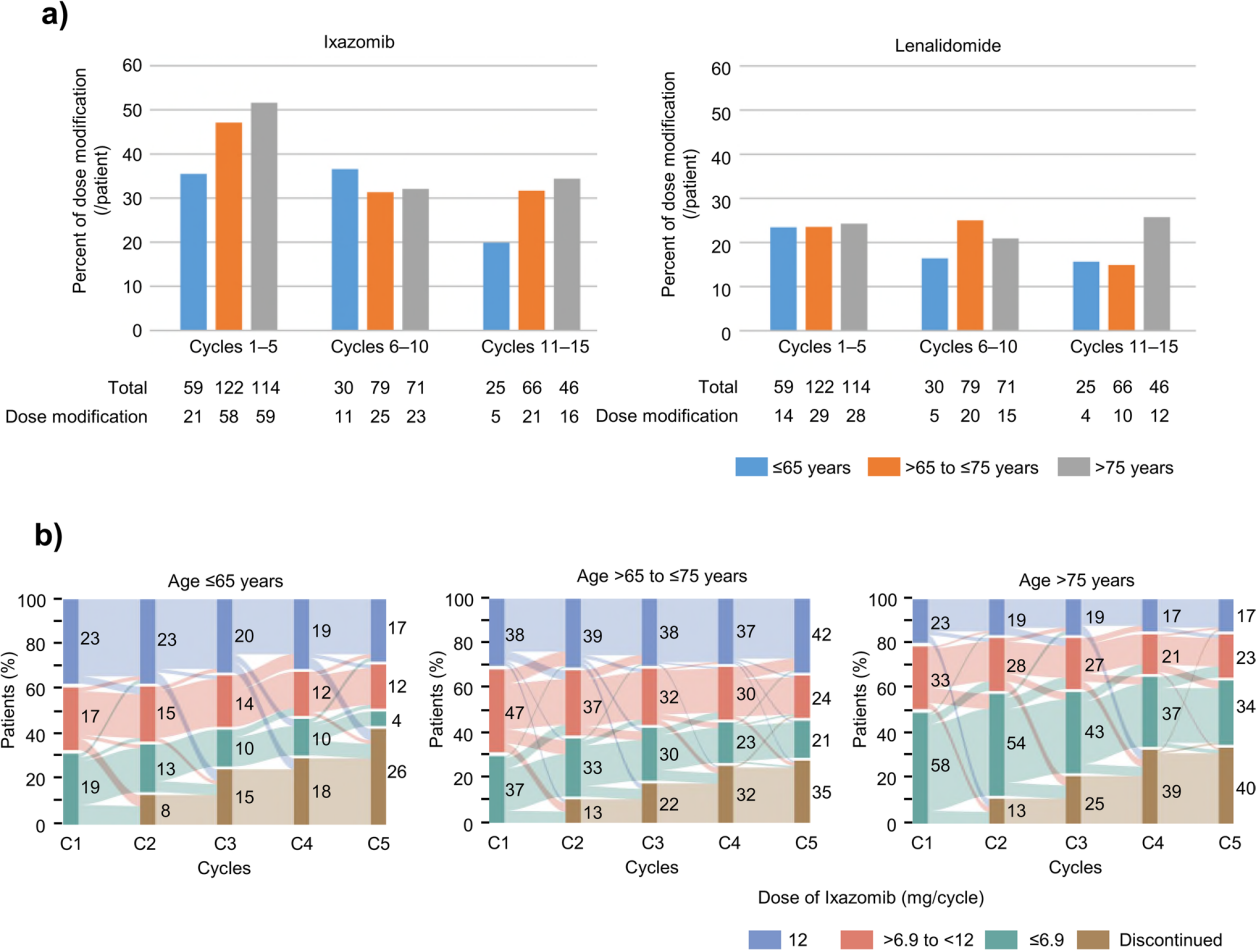
Dose adjustment during treatment cycles, shown by age group. (**a**) Proportion of patients who had a dose adjustment for ixazomib and lenalidomide and (**b**) Sankey diagram showing the initial dose and dose adjustments, with the vertical bars representing various ixazomib dose ranges and the connecting arcs representing the proportion of patients transitioning from one ixazomib dose to the same or different dose in the next treatment cycle

The mean (standard deviation [SD]) and median RDIs were 66.5% (21.1) and 66.7% for ixazomib, 44.7% (22.8) and 44.0% for lenalidomide, and 41.1% (26.6) and 39.9% for dexamethasone. The proportion of patients who had a dose adjustment for ixazomib and lenalidomide are shown in Fig. 1a. Dose adjustment was more frequent among patients aged > 75 years, especially between cycles 1 and 5. Moreover, a higher proportion of patients aged > 75 years tended to start at a lower dose compared with patients aged ≤ 65 years or > 65– ≤ 75 years (Fig. 1b).

### Effectiveness

**Fig. 2:**
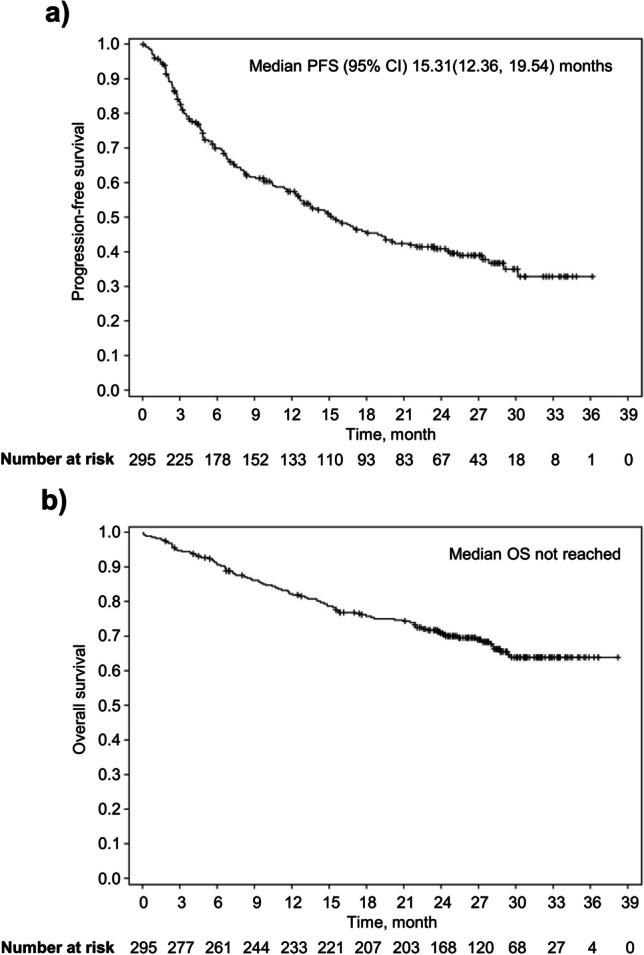
Kaplan–Meier curves of (**a**) progression-free survival (PFS) and (**b**) overall survival (OS) in the full analysis set (*N* = 295). Median OS was not reached. CI, confidence interval

After a median follow-up of 25 months, median PFS was 15.3 (95% CI 12.4–19.5) months (Fig. 2a). The PFS rates at 12 and 24 months were 57.0% (95% CI 51.0–63.0%) and 41.0% (95% CI 35.0–47.0%), respectively. Median OS was not estimable (Fig. 2b). The OS rates were 82.0% (95% CI 77.0–86.0%) and 71.0% (95% CI 65.0–76.0%) at 12 and 24 months, respectively.

**Fig. 3:**
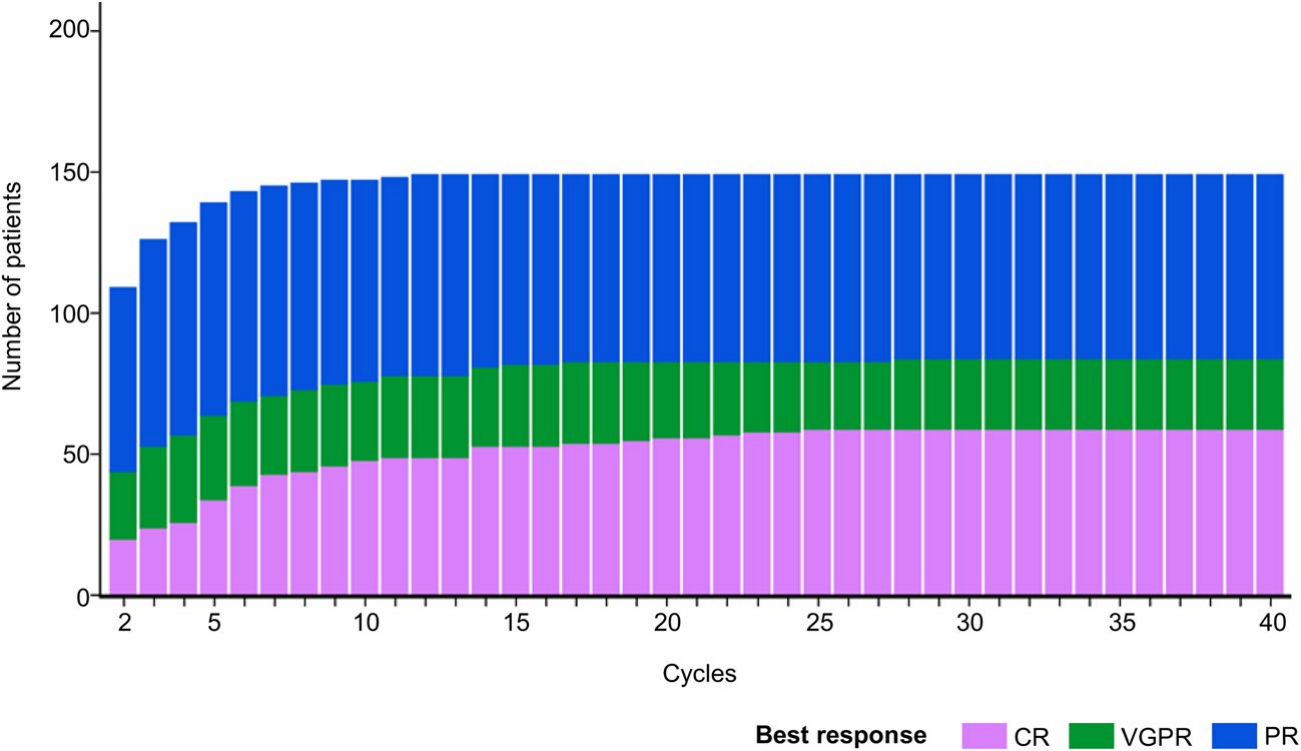
Cumulative best response over time in the full analysis set. CR, complete response; PR, partial response; VGPR, very good partial response

The ORR was 53.9%, and 31.5% of patients (*n* = 93) had ≥ VGPR, with the CR rate being 23.1% (*n* = 68; Fig. 3). Median DOT during this study was 246 (range 1–1108) days. Median TTNT was 13.2 (95% CI 11.1–15.1) months and median DOR was 29.7 (95% CI 23.4–not reached) months (Supplementary Fig. S1).

### Effectiveness by subgroups

**Fig. 4:**
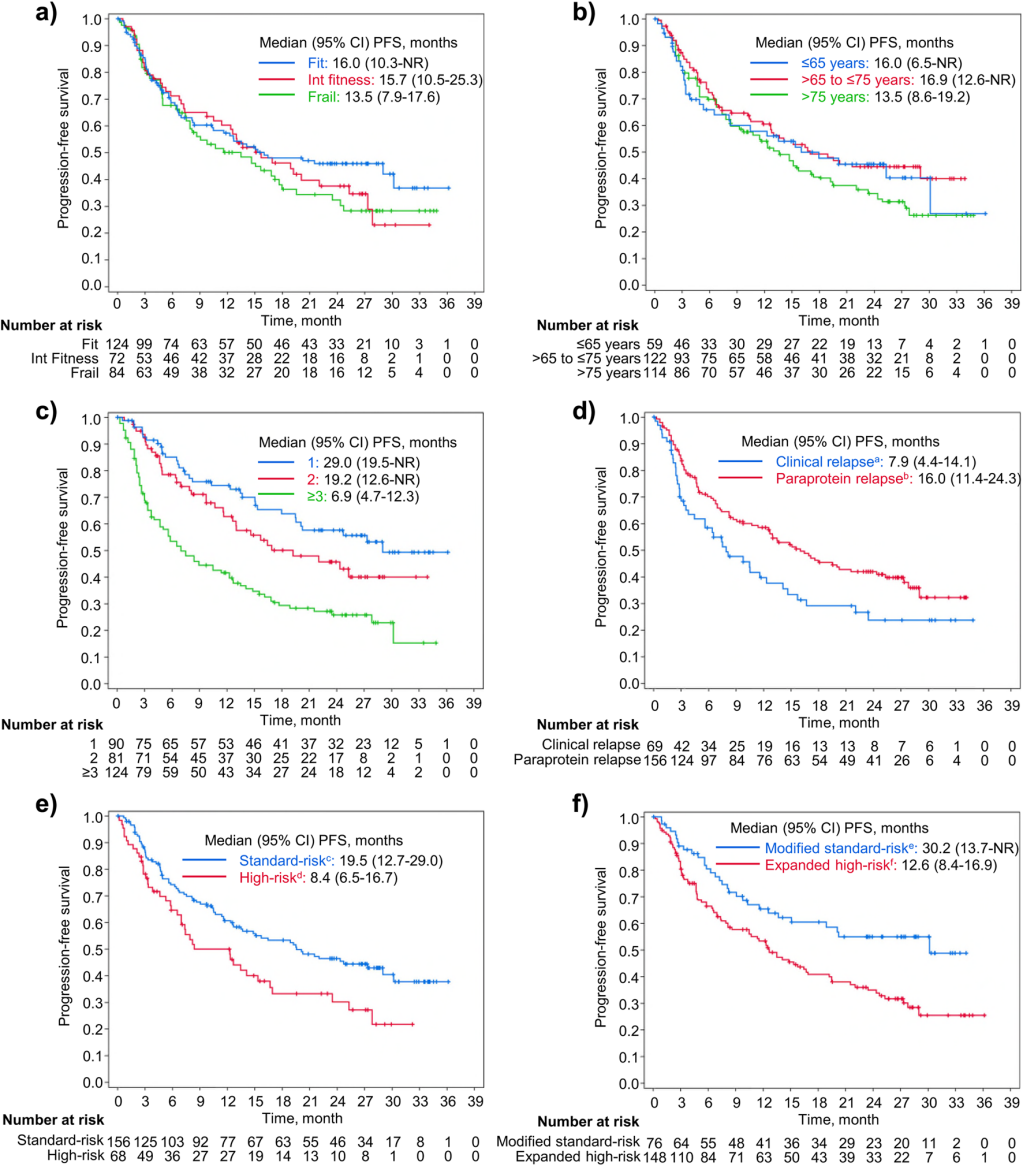
Progression-free survival (PFS) by subgroup according to (**a**) IMWG frailty score, (**b**) age, (**c**) number of prior regimens, (**d**) baseline disease status, (**e**) cytogenetic risk and (**f**) modified cytogenetic risk. ^a^Clinical relapse was defined as disease recurrence with CRAB (i.e., **c**alcium elevation, **r**enal insufficiency, **a**nemia, and **b**one abnormalities) symptoms. ^b^Paraprotein relapse was defined as disease recurrence with elevated M-protein levels but without CRAB symptoms. ^c^Standard-risk was defined as the absence of high-risk cytogenetic abnormalities. ^d^High-risk was defined as the presence of ≥ 1 of t(4;14), t(14;16), or del(17p). ^e^Modified standard-risk was defined as the absence of expanded high-risk cytogenetic abnormalities. ^f^Expanded high-risk was defined as the presence of ≥ 1 of t(4;14), t(14;16), or del(17p), and/or 1q21 gain. CI, confidence interval; IMWG, International Myeloma Working Group; Int, intermediate; NR, not reached

Median PFS was similar across subgroups defined by IMWG frailty score and age. Median PFS by frailty score was 13.5, 15.7, and 16.0 months in the frail, intermediate fitness, and fit subgroups, respectively (Fig. 4a). Median PFS by age was 16.0 months for patients aged ≤ 65 years, 16.9 months for patients aged > 65 to ≤ 75 years, and 13.5 months for those aged ≥ 75 years (Fig. 4b). In patients who had received 1, 2, and ≥ 3 prior regimens, the median PFS was 29.0, 19.2, and 6.9 months, respectively (Fig. 4c). In patients with clinical relapse and paraprotein relapse, the median PFS was 7.9 and 16.0 months, respectively (Fig. 4d). Median PFS was 8.4 months in patients with high-risk cytogenetic abnormalities and 19.5 months in patients with standard-risk cytogenetic abnormalities (Fig. 4e). Median PFS was 12.6 months in patients with expanded high-risk cytogenetic abnormalities and 30.2 months patients with modified standard-risk cytogenetic abnormalities (Fig. 4f).

**Fig. 5:**
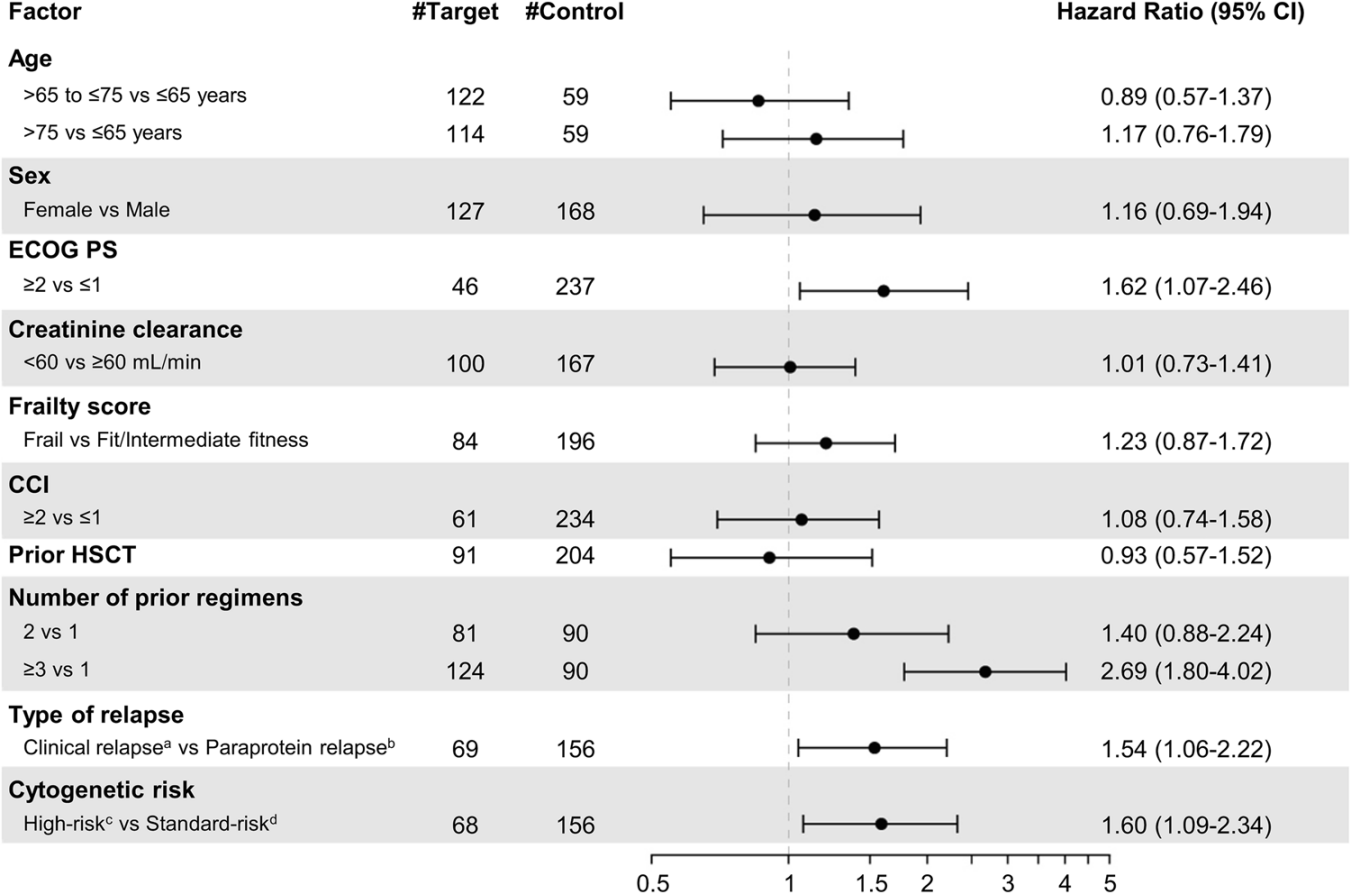
Forest plot of progression-free survival (PFS) to determine the association of patient baseline factors of interest with PFS with IRd treatment. A univariate Cox proportional hazard model was used to estimate the hazard ratios for risk of PFS. ^a^Clinical relapse was defined as disease recurrence with CRAB (i.e., **c**alcium elevation, **r**enal insufficiency, **a**nemia, and **b**one abnormalities) symptoms. ^b^Paraprotein relapse was defined as disease recurrence with elevated M-protein levels but without CRAB symptoms. ^c^High-risk was defined as the presence of ≥ 1 of t(4;14), t(14;16), or del(17p). ^d^Standard-risk was defined as the absence of high-risk cytogenetic abnormalities. CCI, Charlson Comorbidity Index; CI, confidence interval; ECOG PS, Eastern Cooperative Oncology Group performance status; HSCT, hematopoietic stem-cell transplantation; IRd, ixazomib + lenalidomide + dexamethasone

**Fig. 6:**
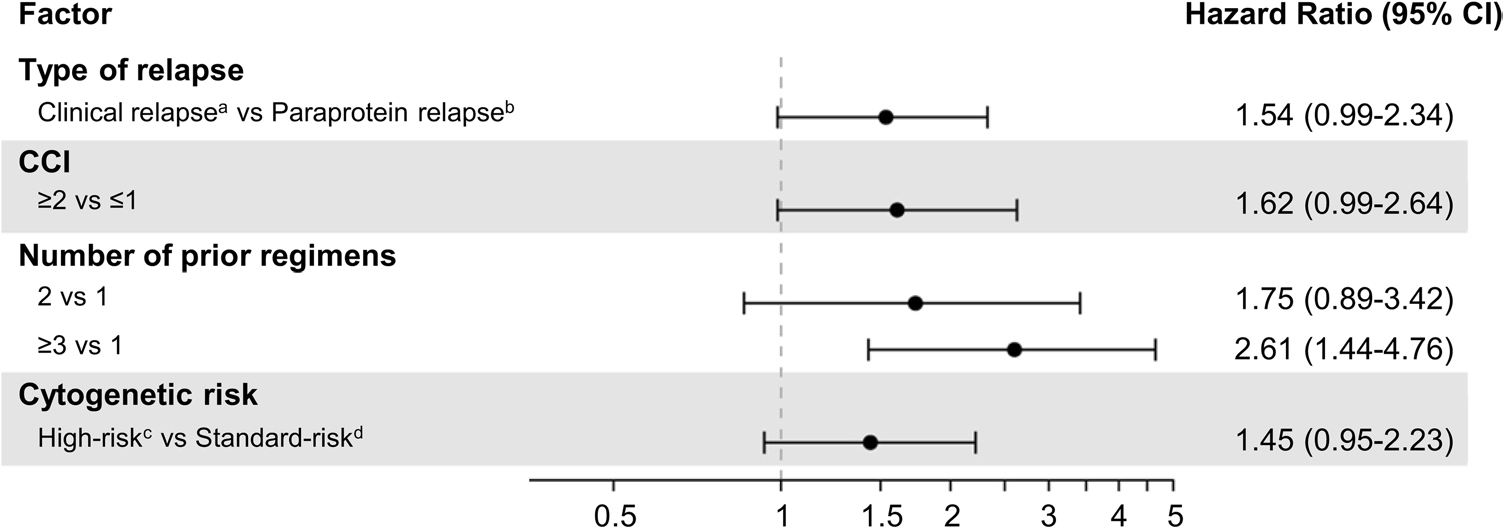
Forest plot of progression-free survival (PFS) to determine the association of patient baseline factors of interest with PFS with IRd treatment. A multivariate Cox proportional hazard model was used to estimate the hazard ratios for risk of PFS. ^a^Clinical relapse was defined as disease recurrence with CRAB (i.e., **c**alcium elevation, **r**enal insufficiency, **a**nemia, and **b**one abnormalities) symptoms. ^b^Paraprotein relapse was defined as disease recurrence with elevated M-protein levels but without CRAB symptoms. ^c^High-risk was defined as the presence of ≥ 1 of t(4;14), t(14;16), or del(17p). ^d^Standard-risk was defined as the absence of high-risk cytogenetic abnormalities. CCI, Charlson Comorbidity Index; CI, confidence interval; IRd, ixazomib + lenalidomide + dexamethasone

Univariate Cox regression analysis identified many factors that were significantly associated with a shorter PFS (Fig. 5), namely: high-risk cytogenetic abnormalities (vs standard-risk; HR = 1.60, 95% CI 1.09–2.34), clinical relapse (vs paraprotein relapse; HR = 1.54, 95% CI 1.06–2.22), ECOG performance status ≥ 2 (vs 0–1; HR = 1.62, 95% CI 1.07–2.46), and number of prior regimens ≥ 3 (vs 1; HR = 2.69, 95% CI 1.80–4.02). Multivariate COX regression analysis found a significant association between a shorter PFS and ≥ 3 prior regimens (vs 1; HR = 2.61, 95% CI 1.44–4.76), but not with high-risk chromosomal abnormalities (vs standard-risk; HR = 1.45, 95% CI 0.95–2.23; Fig. 6).

### Health-related quality of life

There were no considerable changes in overall health score or HRQoL during treatment (Supplementary Fig. S2).

### Minimal residual disease negativity

MRD was measured using the SRL-flow method [19] in 30 patients with CR (i.e., in < 50% of patients achieving CR; Supplementary Table S5). As such, MRD negativity was studied in a small portion (10.2%) of the overall population. In this subgroup, the proportion of patients achieving MRD < 10^–4^ was 73.3%, those achieving MRD < 10^–5^ was 56.7%, and patients with MRD < 10^–6^ were 50.0%.

### Safety

TEAEs of any grade were reported in 84.4% of patients, grade ≥ 3 TEAEs in 58.0%, serious TEAEs in 32.5%, TEAEs leading to discontinuation in 24.7%, and TEAEs leading to death in 8.1% (Table 2). IRd treatment-related adverse events occurred in 75.6% of patients and adverse drug reactions (ADRs) in 71.5% of patients. The most common TEAEs of any grade were thrombocytopenia (28.8%), neutropenia (28.8%), diarrhea (27.1%), and leukopenia (25.8%). The most common grade ≥ 3 TEAEs were thrombocytopenia (21.4%), neutropenia (16.3%), leukopenia (11.9%), and anemia (7.5%; Table 2). The most common ADRs were thrombocytopenia (25.8%), diarrhea (25.4%), neutropenia (24.4%), and thrombocytopenia (22.0%). The most common TEAEs leading to treatment discontinuation were diarrhea (4.4%), platelet count decreased (3.4%), peripheral neuropathy (2.4%), rash (1.7%), and pneumonia (1.4%). No new safety concerns were identified.

**Table 2:** Treatment-emergent adverse events in the safety analysis set

***n*** **(%)**	***N*** = 295	
Any TEAEs	249 (84.4)	
Grade ≥ 3 TEAEs	171 (58.0)	
IRd treatment-related TEAEs	223 (75.6)	
IRd treatment-related grade ≥ 3 TEAEs	137 (46.4)	
ADRs	211 (71.5)	
Grade ≥ 3 ADRs	127 (43.1)	
Serious TEAEs	96 (32.5)	
TEAEs leading to treatment discontinuation	73 (24.7)	
TEAEs leading to death^a^	24 (8.1)	
**TEAEs occurring in ≥ 5.0% patients** **System organ class** **Preferred term**	**Overall**	**Grade ≥ 3**
Blood and lymphatic system disorders		
Anemia	31 (10.5)	22 (7.5)
Leukopenia^b^	76 (25.8)	35 (11.9)
Neutropenia^c^	82 (27.8)	48 (16.3)
Thrombocytopenia^d^	85 (28.8)	63 (21.4)
Gastrointestinal disorders		
Diarrhea	80 (27.1)	16 (5.4)
Constipation	20 (6.8)	-
Nausea	18 (6.1)	-
Vomiting	17 (5.8)	2 (0.7)
Infections and infestations		
Pneumonia	26 (8.8)	18 (6.1)
Nasopharyngitis	16 (5.4)	-
Skin and subcutaneous tissue disorders		
Rash	40 (13.6)	3 (1.0)

*ADR* adverse drug reaction, *IRd* ixazomib + lenalidomide + dexamethasone, *TEAE* treatment-emergent adverse event

^a^TEAEs leading to death included plasma cell myeloma (*n* = 12; 4.1%), cardio-respiratory arrest, pneumonia, pneumonia influenza (*n* = 2; 0.7% each), and cardiac arrest, cardiac failure, cardiac death, death, multiple organ dysfunction syndrome, sepsis, acute kidney injury, interstitial lung disease, and pulmonary amyloidosis (*n* = 1; 0.3% each)

^b^Includes leukopenia and white blood cell count decreased

^c^Includes neutropenia and neutrophil count decreased

^d^Includes thrombocytopenia and platelet count decreased

There were no significant differences between the three age groups in the rate of treatment discontinuation due to TEAEs (Supplementary Fig. S3), with 32% of patients aged ≤ 65 years, 21% of patients aged > 65 to ≤ 75 years, and 21% of those aged ≥ 75 years discontinuing treatment due to TEAEs. The RDI had no impact on the incidence of treatment discontinuation across age groups.

## Discussion

The present real-world study conducted in Japan in patients with RRMM showed that IRd therapy had promising clinical activity and a manageable safety profile, consistent with the findings of the phase III TOURMALINE-MM1 trial [10]. The results of our study are encouraging, especially considering the unfavorable patient disease status/demographic characteristics at treatment initiation.

The median PFS was 15.3 months and ORR and ≥ VGPR were 53.9% and 31.5%, respectively. As expected, outcomes were inferior to results of the TOURMALINE-MM1 trial (median PFS: 20.6 months; ORR: 78.0%; and ≥ VGPR: 48.0%), because a more diverse patient population was included compared with a selected patient population in randomized controlled trials in patients with MM [11]. For example, compared with the TOURMALINE-MM1 trial [10], in this real-world study, there were more patients of advanced age (> 65 years; 80.0% vs 53%), poor renal function (< 60 mL/min; 37.3% vs 22%), poor ECOG performance status (≥ 2; 15.6% vs 5%), more patients with at least three lines of prior treatment (42.1% vs 11%) and more patients who had prior PI (bortezomib/carfilzomib; 76.6%/12.9% vs 69%/ < 1%) or lenalidomide (82.7% vs 12%) therapy. In addition, the present study included patients who were refractory to prior lenalidomide or PI-based therapy, unlike the TOURMALINE-MM1 trial [10]. It is also important to note that the PFS benefits of IRd treatment were observed regardless of frailty or age, confirmed in a Cox regression analysis. These findings are important because the number of elderly/frail patients with RRMM is likely to increase in the future as new drugs become available.

Moreover, the inclusion of older and frail patients could be one of the reasons for the lower ixazomib RDI compared with TOURMALINE-MM1, which had a younger patient population [10]. In the present study, the initial dose of ixazomib tended to be lower in older patients and dose adjustments of ixazomib were numerically more frequent from cycle 1 to cycle 5. This approach may have resulted in favorable PFS in older or frail patients by ensuring tolerability and treatment continuation.

The TOURMALINE-MM1 trial reported consistent PFS benefits with IRd treatment compared with Rd treatment in key prespecified patient subgroups such as age category and the number of prior therapies, the history of prior PI/immunomodulatory therapy and cytogenetic abnormalities [10]. Despite the limitation of this observational study being a single-arm study, our results also suggest that an acceptable PFS can be expected with IRd treatment in certain subgroups. Comparison of patient subgroups based on the number of prior therapies showed that the median PFS was either longer or comparable with that reported in TOURMALINE-MM1 (1, 2, and ≥ 3 lines of prior treatment: 29.0, 19.2, and 6.9 months vs 20.6 and 17.5 months and not estimable, respectively) [10], especially in patients with 1 or 2 prior therapies.

Several other studies have investigated the benefit of IRd treatment in patients with MM in a real-world setting [20–30]. Our results add to the body of evidence from these studies, which have shown the effectiveness of IRd in a broad range of patients, including frail, older, and heavily pre-treated patients and those with advanced disease [21, 23, 25, 28–30]. Of these studies, seven are fully published [22, 23, 26–30]; the other studies have only interim data available (INSIGHT MM [31], UVEA-IXA [21], and REMIX [25]). INSIGHT MM is the largest of these studies: 4,200 patients with newly diagnosed MM or RRMM have been enrolled from 15 countries worldwide, including the United States and countries in Europe, Asia, the Middle East, and Latin America, and the planned follow-up is a minimum of 5 years [31]. In the Czech study in 344 patients with RRMM, which was a nonrandomized prospective two-arm study, IRd was superior to Rd, with a median PFS of 17.5 months versus 11.5 months (*p* = 0.005) after a median follow-up of 20.8 and 15.5 months, respectively. This PFS advantage translated into improved OS for patients treated with IRd (median OS 36.6 vs 26.0 months with Rd; *p* = 0.008) [22]. Notably, patients with 1–3 disease relapses had a median PFS of 23.1 months after treatment with IRd, versus 11.6 months after Rd treatment (*p* = 0.001). The PFS and OS benefits of IRd were sustained in the overall population (median PFS 17.5 vs 12.5 months with Rd; *p* = 0.013 and median OS 40.9 vs 27.1 months; *p* = 0.001), with better outcomes in patients with 1–3 disease relapses (median PFS 22.3 vs 12.7 months; *p* = 0.003 and median OS 51.7 vs 27.8 months; *p* < 0.001) over a prolonged follow-up of 28.5 months [32]. In the noncomparative Slovakian (*n* = 106) [23], Korean (*n* = 60) [30], and European (*n* = 155) [26] studies in patients with RRMM, the ORR was 74.0–85.0%, and although median OS was not reached in all three studies, the median PFS was 25.9–43.0 months. In the INSIGHT-RMG pooled analysis (*n* = 263), 56.3% of patients received IRd in ≥ third line. The ORR was 73.0%, median PFS 21.2 months, TTNT 33.0 months, and median OS was not reached. In two retrospective database analyses comparing PI-triplet regimens with an RD backbone in US, among patients receiving IRd (*n* = 168 [27] and 154 [28] for IRd, respectively) approximately 40.0% were ≥ 75 years of age and 59.0–63.0% received IRd as ≥ third line of therapy. IRd was more likely to be recommended in patients aged ≥ 75 years [28] and those with high-risk cytogenetic abnormalities and as a late-line therapy [27]. It was associated with lower risk of treatment discontinuation and longer TTNT, especially in intermediate/frail patients [27, 28]. Based on the findings of real-world studies, including the current study, IRd therapy may prove to be beneficial in clinical practice for patients with unfavorable disease status/demographic characteristics, particularly for those who are frail and often have trouble continuing treatment.

Cytogenetic abnormalities in patients with MM, such as t(4;14), t(14;16), del(17p), and 1q21 gain, are indicative of poor prognosis [33]. The proportions of patients with high-risk and individual cytogenetic abnormalities in this study were generally higher than those in the TOURMALINE-MM1 trial (proportion of patients with expanded high-risk: 43.1%; del(17p): 10.0%; 1q21 gain: 22.2%) [34, 35]. In the present study, the median PFS in patients with high-risk cytogenetic abnormalities was shorter than in patients with standard-risk cytogenetic abnormalities. On the other hand, in the TOURMALINE-MM1 trial, which had a higher proportion of patients with early line of therapy (1, 2, and 3 prior regimens were 62%, 27%, and 11%, respectively), the median PFS for patients with high-risk cytogenetic abnormalities was comparable with the median PFS for patients with standard-risk cytogenetic abnormalities (21.4 vs 20.6 months, respectively) [10]. One possible explanation for a difference in PFS between these patient subgroups in our study was that patients with high-risk cytogenetic abnormalities were more heavily pre-treated. The proportion of patients with 1, 2, and ≥ 3 prior regimens among patients with high-risk cytogenetic abnormalities were 23.5%, 27.9%, and 48.5%, respectively. In contrast, the proportion of patients with 1, 2, and ≥ 3 prior regimens among patients with standard-risk cytogenetic abnormalities were 32.7%, 28.8%, and 38.5%, respectively. It is possible that patients with high-risk cytogenetic abnormalities had a higher number of prior treatments due to their tendency to relapse earlier and have poorer outcomes. The median PFS was 29.0 months for patients with one line of therapy, while it was 6.9 months for those with ≥ 3 lines of treatment. These imbalances in the background of prior treatment history between patients with high-risk cytogenetics and standard-risk cytogenetics in our study may have influenced the results of cytogenetic abnormalities subgroup analysis. The results of the multivariate COX regression analysis support this hypothesis, as it indicated that high-risk cytogenetic abnormalities themselves are not an independent factor affecting PFS, but the number of prior treatment regimens was an independent variable affecting PFS.

The safety profile of IRd in this study was similar to that reported in the TOURMALINE-MM1 trial and a phase 2 trial in Japanese patients with RRMM [10, 36], and no new safety concerns were identified. Hematologic TEAEs were more frequently reported than nonhematologic TEAEs. Among grade ≥ 3 TEAEs with ≥ 5.0% incidence, the most common IRd-related hematologic TEAEs included thrombocytopenia in 57 patients (19.3%), neutropenia in 45 patients (15.3%), leukopenia in 32 patients (10.8%), and anemia in 17 patients (5.8%). Commonly reported IRd-related nonhematologic grade ≥ 3 TEAEs included diarrhea and pneumonia in 15 patients each (5.1%). Safety results were similar to those from the Czech study [22, 32], but differed from studies of Korean and European patients with RRMM, where grade ≥ 3 nonhematologic AEs, such as infections, skin rash, gastrointestinal toxicities, and peripheral neuropathy were more common than hematologic AEs [26, 30]. The most common TEAEs leading to discontinuation in our study were diarrhea, thrombocytopenia, peripheral neuropathy, rash, and pneumonia. The management of these TEAEs is important in clinical practice to ensure patient adherence and therefore long-term treatment.

Long-term PI-based therapy has been shown to improve outcomes in MM [37]. Cancer patients prefer oral over intravenous administration for reasons of convenience, perceived efficacy and past experience [38]. Since the IRd triplet regimen is given orally, patients may be more likely to adhere to IRd treatment than PI agents administered by injection, thus improving the possibility for long-term treatment. Future studies of IRd should examine treatment adherence and its effect on duration of treatment.

This real-world study has a few limitations. Firm conclusions about the benefits of ixazomib, including findings in subgroups, cannot be made because of the single-arm study design and the small number of patients in our study. Furthermore, due to the collection of data through the electronic data capture system, we were unable to determine what proportion of patients enrolled in this study were refractory to prior lenalidomide or PI-based therapy, which could limit interpretation of our findings. Thus, further studies in a larger patient population are required to confirm these findings.

## Conclusion

In this real-world study of a diverse population with RRMM in Japan, the oral IRd triplet regimen was an effective and tolerable treatment option. Given that these patients were relatively frail, older, and more heavily treated than the RRMM patients included in IRd clinical trials, these results are particularly encouraging for patients with unfavorable disease status/demographic characteristics.

### Supplementary Information

Below is the link to the electronic supplementary material.

Supplementary file1 (DOCX 191 KB)

## Data Availability

The datasets, including the redacted study protocol, redacted statistical analysis plan, and individual participants’ data supporting the results reported in this article, will be made available within 3 months from initial request, to researchers who provide a methodologically sound proposal. The data will be provided after its de-identification, in compliance with applicable privacy laws, data protection and requirements for consent and anonymization.
