# Supplementary material for: A prospective, multicenter, observational study of ixazomib plus lenalidomide-dexamethasone in patients with relapsed/refractory multiple myeloma in Japan
Source: Ann Hematol. 2023 Sep 11;103(2):475–88. doi: 10.1007/s00277-023-05428-7 (PMC10798923; doi:10.1007/s00277-023-05428-7)

**A Prospective, Multicenter, Observational Study of Ixazomib plus Lenalidomide-Dexamethasone in Patients with Relapsed/Refractory Multiple Myeloma in Japan**

**Supplementary Materials**

**Journal:** Annals of Hematology

**Authors:** Yuichi Horigome^1^, Masaki Iino^2^, Yoriko Harazaki^3^, Takahiro Kobayashi^4^, Hiroshi Handa^5^, Yasushi Hiramatsu^6^, Taiga Kuroi^7^, Kazuki Tanimoto^8^, Kosei Matsue^9^, Masahiro Abe^10^, Tadao Ishida^11^, Shigeki Ito^12^, Hiromi Iwasaki^13^, Junya Kuroda^14^, Hirohiko Shibayama^15^, Kazutaka Sunami^16^, Hiroyuki Takamatsu^17^, Hideto Tamura^18^, Toshiaki Hayashi^19^, Kiwamu Akagi^20^, Takahiro Maeda^21^, Takahiro Yoshida^22^, Ikuo Mori^22^, Tomohiro Shinozaki^23^, Shinsuke Iida^24^

**Corresponding author:** Shinsuke Iida, Department of Hematology and Oncology, Nagoya City University Institute of Medical and Pharmaceutical Sciences, 1, Kawasumi, Mizuho-cho, Mizuho-ku, Nagoya, Aichi 467-8601, Japan. Tel: +81-52-853-8738; Fax: +81-52-853-8740; Email: [iida@med.nagoya-cu.ac.jp](mailto:iida@med.nagoya-cu.ac.jp); ORCID iD: 0000-0002-4951-960X

## Supplementary Methods

### Definitions of pre-specified endpoints

Progression-free survival (PFS) was defined as the time from the first ixazomib plus lenalidomide and dexamethasone dose (hereafter known as IRd) to confirmed progressive disease or death. Overall survival (OS) was defined as the time from the first IRd dose to confirmed death; time to next treatment (TTNT) as the time from the first IRd dose to the start of next treatment or confirmed death, whichever is earlier; duration of response (DOR) as the time from the date of first documentation of response to the date of first documented progression; and overall response rate (ORR) as the proportion of patients who achieve a best response of partial response (PR) or better according to the International Myeloma Working Group (IMWG) criteria [33].

### Safety and tolerability

Safety and tolerability were assessed by recording any treatment-emergent adverse events (TEAEs), defined as any untoward medical occurrence in a study subject, arising or worsening after the start of IRd therapy until 30 days after the last dose of IRd therapy or the start of the next treatment, whichever was earlier. An adverse drug reaction (ADR) was any untoward medical occurrence that was considered related to ixazomib treatment. TEAEs were graded using the NCI Common Terminology Criteria for Adverse Events Japanese version 4.03. TEAEs were classified by using the preferred terms (PT) and system organ classes (SOC) in the Japanese version of the Medical Dictionary for Regulatory Activities (MedDRA/J) version 23.0.

### Genomic analysis

To conduct genomic analyses, bone marrow samples were collected at the start of IRd therapy for cytogenetic testing and at initial assessment of complete response (CR; i.e., very good partial response or better [≥VGPR]) for minimal residual disease (MRD) measurement. Cytogenetic testing was done at the central laboratory; CD138-positive cells were isolated and the proportion of cells positive for t(4;14), t(11;14), t(14;16), 1q21 gain, or del(17p) were measured. Cut-off levels were 5% positive cells for del(17p) and 3% for t(4;14), t(11;14), t(14;16), and 1q21 gain. The threshold for TOURMALINE-MM1 cut-off values was used to determine these levels. The next-generation sequencing results at the start of IRd therapy were used to determine MRD at the time of CR. In patients with ≥VGPR, MRD measurements were performed by single-tube 8-color multiparameter flow cytometry (SRL-flow) [19] when a physician and the patient requested MRD measurements.

### Patient-reported quality of life assessments

The European Organisation for Research and Treatment of Cancer Quality of Life Questionnaire-Core 30 module (EORTC QLQ-C30) comprises five functional scales (physical, role, cognitive, emotional, and social), three symptom scales (fatigue, pain, and nausea/vomiting), a global health status/quality of life (QoL) scale, and six single-item scales (dyspnea, loss of appetite, insomnia, constipation, diarrhea, and perceived financial impact of the disease) [34]. Scale scores range from 0 to 100, with higher scores representing a better health state for the functional scores and global health status/QoL and lower scores representing a better health state for the symptom scores.

The myeloma-specific module EORTC QLQ-MY20 consists of a 20-item questionnaire grouped into four scales: disease symptoms, treatment adverse effects, body image, and future perspective [34]. Scale scores range from 0 to 100, with higher scores representing higher levels of symptomatology or problems for disease symptoms and treatment adverse effects and better outcomes for body image and future perspective.

## Supplementary Table S1.

Complete inclusion and exclusion criteria

| **Inclusion criteria** | **Exclusion criteria** |
| --- | --- |
|  | |
| - Men and women aged ≥20 years at the time of enrollment - Patients with RRMM Patients who are scheduled to start IRd therapy - Patients who can provide written informed consent of their own free will before the start of study treatment - Patients who are judged by the principal investigator(s) to have the faculty to understand and comply with the requirements of the study | - Female patients who are nursing or pregnant - Patients who have been previously treated with ixazomib - Patients with hypersensitivity to any of the components of IRd therapy, their analogs or excipients - Patients with another active malignancy (i.e., synchronous active malignancy or previous malignancy with a disease-free period <5 years, except for patients with carcinoma *in situ* [intraepithelial carcinoma] or intramucosal carcinoma judged to be cured by topical treatment) - Patients who are not registered with, or comply with, the guidelines of the lenalidomide management program (RevMate®) - Patients who, in the judgement of the principal investigator(s), are considered to be unsuitable for enrolment into the study |

IRd, ixazomib + lenalidomide + dexamethasone; RRMM, relapsed/refractory multiple myeloma.

## Supplementary Table S2.

Cross table of cytogenetic abnormalities prior to ixazomib-lenalidomide-dexamethasone therapy

| **Category, *n* (%)^a^** | **Overall (*N* = 224)** | | | | |
| --- | --- | --- | --- | --- | --- |
|  | **Single** | **Double** | | | **Triple** |
|  |  | t(4;14) or  t(14;16) | 1q21 gain | del(17p) |  |
| t(4;14) or t(14;16) | 9 (4.0) | - | 15 (6.7) | 2 (0.9) | 14 (6.3) |
| 1q21 gain | 80 (35.7) | 15 (6.7) | - | 15 (6.7) | 14 (6.3) |
| del(17p) | 13 (5.8) | 2 (0.9) | 15 (6.7) | - | 14 (6.3) |

^a^Cut-off levels were 5% positive cells for del(17p) and 3% for t(4;14), t(14;16), and 1q21 gain.

## Supplementary Table S3.

Ten most common drug regimens at any line of treatment prior to ixazomib-lenalidomide-dexamethasone therapy

| **Prior regimen, *n* (%)^a^** | ***N* = 295** |
| --- | --- |
| Lenalidomide + dexamethasone | 155 (52.5) |
| Bortezomib + dexamethasone | 109 (36.9) |
| Bortezomib + lenalidomide + dexamethasone | 59 (20.0) |
| Melphalan | 35 (11.9) |
| Bortezomib + dexamethasone + cyclophosphamide | 34 (11.5) |
| Bortezomib + prednisolone + melphalan | 31 (10.5) |
| Pomalidomide + dexamethasone | 30 (10.2) |
| Bortezomib | 20 (6.8) |
| Lenalidomide | 19 (6.4) |
| Carfilzomib + dexamethasone | 18 (6.1) |
| Prednisolone + melphalan | 18 (6.1) |

^a^Patients may have received >1 prior treatment regimen; *n* (%) denotes the number and percentage of patients, not the number and percentage of previous therapies.

## Supplementary Table S4.

Ten most common drug regimens received immediately prior to ixazomib-lenalidomide-dexamethasone therapy

| **Prior regimen, *n* (%)** | ***N* = 295** |
| --- | --- |
| Lenalidomide + dexamethasone | 90 (30.5) |
| Bortezomib + lenalidomide + dexamethasone | 29 (9.8) |
| Bortezomib + dexamethasone | 28 (9.5) |
| Lenalidomide | 15 (5.1) |
| Pomalidomide + dexamethasone | 12 (4.1) |
| Carfilzomib + dexamethasone | 11 (3.7) |
| Lenalidomide + dexamethasone + elotuzumab | 11 (3.7) |
| Melphalan | 11 (3.7) |
| Lenalidomide + dexamethasone + daratumumab | 9 (3.1) |
| Bortezomib | 7 (2.4) |
| Carfilzomib + lenalidomide + dexamethasone | 7 (2.4) |

## Supplementary Table S5.

Proportion of patients with complete response who achieved minimal residual disease negativity

|  | **SRL-flow method** |
| --- | --- |
| Patients with CR, *n* (%) | 68 (23.1) |
| Patients with MRD negative cells, *n* | 30 |
| Proportion of MRD negative cells, *n* (%) |  |
| <10^–4^ | 22 (73.3) |
| 95% CI | 54.1, 87.7 |
| <10^–5^ | 17 (56.7) |
| 95% CI | 37.4, 74.5 |
| <10^–6^ | 15 (50.0) |
| 95% CI | 31.3, 68.7 |

CI, confidence interval CR, complete response; MRD, minimal residual disease; SRL, single-tube 8-color multiparameter flow cytometry.

## Supplementary Fig. S1

Kaplan–Meier analysis of duration of response (DOR) from the start of treatment.


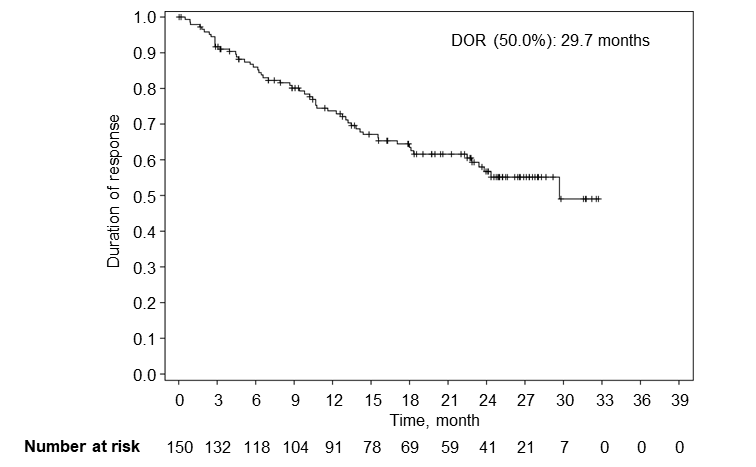


## Supplementary Fig. S2

Time plot of mean ± standard deviation (SD) changes in health-related quality of life for (a) EORTC QLQ-C30 global health status score, (b) EORTC QLQ-C30 pain score, (c) EORTC QLQ-C30 diarrhea score, and (d) EORTC QLQ-MY20 disease symptom score. EORTC, European Organisation for Research and Treatment of Cancer; QLQ-C30; Quality of Life Questionnaire-Core 30 module; QLQ-MY20, Multiple Myeloma Module Quality of Life Questionnaire.


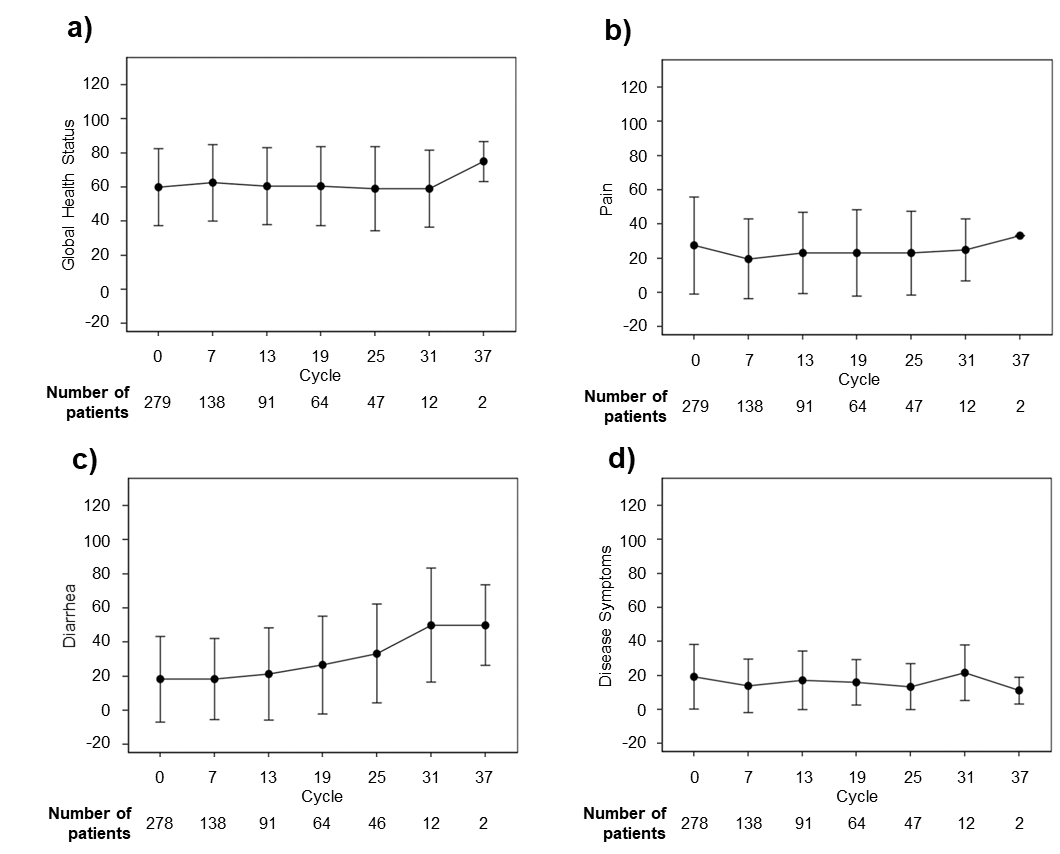


## Supplementary Fig. S3

Adverse events leading to discontinuation according to age group and ixazomib relative dose intensity.


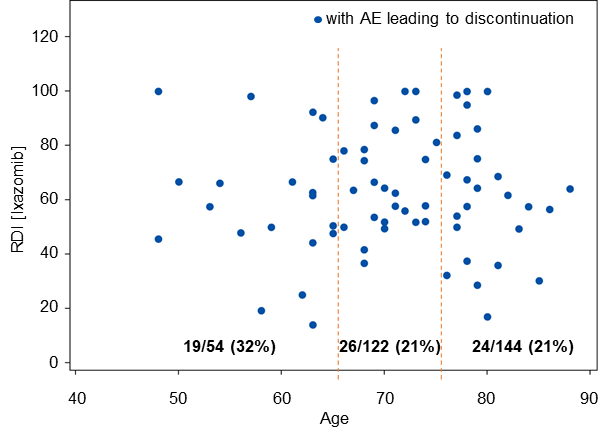

Supplement: Supplementary file 1 — Supplementary file1 (DOCX 191 KB) [file 277_2023_5428_MOESM1_ESM.docx]
